# Optical characterization of endoscopes for StoneSense™ compatibility

**DOI:** 10.1007/s00345-026-06845-9

**Published:** 2026-11-02

**Authors:** Nicola Nannola, Carlos Gonzalez-Gonzalez, Federico Zorzi, Pietro Scilipoti, Adèle Martin-Illac, Frédéric Panthier, Marie Chicaud, Steeve Doizi, Iliya Yaroslavsky, Anastasia Kovalenko, James Childs, Igor Perchuk, Olivier Traxer

**Affiliations:** 1 Service d’urologie, Tenon Hôpital, 4 Rue de la Chine, 75020 Paris, France; 2 GRC20 - Endolase lab, Sorbonne University, PIMM-Arts et Métiers Paris Tech, F-75013 Paris, France; 3 PIMM, UMR 8006 CNRS-Arts et Métiers ParisTech, 151 bd de l’Hôpital, F-75013 Paris, France; 4 Department of Urology, Poitiers University Hospital, 2 Rue de la Milétrie, 86000 Poitiers, France; 5 Department of Urology, University of Modena and Reggio Emilia, 41121 Modena, Italy; 6 Department of Experimental Oncology/Unit of Urology, URI, IRCCS Ospedale San Raffaele, Milan, Italy; 7 Vita-Salute San Raffaele University, Milan, Italy; 8 Research and Development, IPG Medical, Marlborough, MA USA; 9 Service d’Urologie, Centre Hospitalier Universitaire de Limoges, 2 avenue Martin Luther King, 87000 Limoges, France

**Keywords:** Flexible ureteroscopy, Cystoscopy, Stone recognition, Spectrophotometry, Laser lithotripsy, StoneSense, Optical compatibility, Pulse-width modulation

## Abstract

**Background and objective:**

The StoneSense Module (SSM), integrated within IPG laser lithotripsy systems, uses endoscopic white-light illumination for real-time stone-tissue discrimination, enabling conditional laser firing only in the presence of a confirmed calculus. SSM performance depends on the endoscope’s optical output falling within defined thresholds. We aimed to characterize commercially available flexible ureteroscopes and cystoscopes against predefined SSM compatibility criteria.

**Methods:**

Twenty device-videobox combinations (15 flexible ureteroscopes, 4 cystoscopes, 1 semirigid ureteroscope; 7 manufacturers) with complete StoneSense-processed band data were included. Total power and spectral band power (Bands 1–3) were measured at three brightness levels in free-space configuration (no fiber inserted), and temporal profile was classified by oscilloscopy. Per-fiber band power was modeled from these free-space measurements, not measured with the fiber in the working channel. Predicted SSM band-power compatibility was assessed per device-brightness-fiber combination against predefined thresholds, with a minimum PWM frequency of 2 kHz as the temporal criterion.

**Results:**

All 20 devices exhibited continuous-wave (CW) emission, conferring unconditional temporal SSM compatibility. Following recalibration of the SSM reference thresholds, the 200 µm and 365 µm fibers were predicted to meet SSM band power thresholds at medium/maximum brightness for 18 of the 20 devices, with the Karl Storz Flex XC and the Olympus CYF-VH cystoscope as the two exceptions at both fiber sizes. Additional saturation, restricted to the 550 µm and 940 µm fibers, was identified for six further devices (Table 1). Four Olympus platforms showed modeled sub-threshold power at minimum brightness. Four illumination archetypes were identified: blue-pump LED, red-shifted LED, Olympus intermediate LED, and LED-phosphor.

**Conclusions:**

The 200 µm and 365 µm fibers provide the broadest predicted compatibility (18/20 platforms), with the Flex XC and CYF-VH as the exceptions. Predicted incompatibilities are otherwise device-architecture-driven and manageable through fiber and brightness selection. A device-level compatibility reference matrix based on modeled estimates is provided.

**Supplementary Information:**

The online version contains supplementary material available at https://doi.org/10.1007/s00345-026-06845-9.

## Introduction

Urinary stone disease affects 10–15% of the global population over a lifetime, with rising prevalence [1, 2]. Flexible ureteroscopy (fURS) with laser lithotripsy is the reference standard for minimally invasive stone treatment [3, 4, 22], with holmium:YAG and thulium fiber laser as the dominant energy platforms [5–7]. The technique requires careful endoscopic navigation, as inadvertent laser activation against urothelial mucosa—often triggered by reduced visibility from stone dust, blood, and scope deflection—may cause mucosal perforation or delayed ureteral stricture [8, 9, 21]. A real-time stone-tissue discrimination system capable of conditionally inhibiting laser firing would address this safety need.

Stone-tissue optical discrimination has been explored through several modalities, including autofluorescence spectroscopy, which exploits calculi’s intrinsic fluorescence [11] and has enabled automatic laser-trigger stone recognition [12–15]. Diffuse reflectance spectroscopy (DRS) using broadband white-light backscatter, first demonstrated by Lange et al. [10], has since been implemented clinically (93% sensitivity/specificity for stone/tissue detection [16]) and entered first-in-human evaluation with automatic laser blocking [17]. The DRS system reported by Korneva et al. [16] shares SSM’s general diffuse-reflectance approach: illumination is delivered via the endoscope and detection occurs through the treatment laser fiber itself — the basis for the compatibility characterization performed here.

The StoneSense Module (SSM), integrated within the LUROS and Gemini systems (IPG Medical Corporation, Marlborough, MA, USA), performs per-pulse stone-tissue classification from this backscattered signal. Pre-clinical evaluation of this laser system’s emission-control performance is reported separately [24, 25]; this manuscript addresses device-compatibility characterization only. Stone-tissue discrimination sensitivity and specificity across stone compositions and colors are being characterized separately, in an ongoing in vitro study, for the compatible device-fiber combinations identified here. At the time of this study the SSM had been FDA-cleared but had not yet received CE marking; this manuscript is part of its technical characterization, not a clinical validation. SSM performance depends on the endoscope’s optical output falling within defined bounds for spectral composition, total power, and temporal profile; out-of-bound parameters risk insufficient signal, detector saturation, or classification errors.

Contemporary endoscopes employ xenon arc lamp, LED, or LED-phosphor illumination, producing substantial inter-device variation in spectra, power output, and temporal emission [18, 19]. Pulse-width modulation (PWM), used by some platforms for brightness regulation, introduces temporal fluctuations below 2 kHz, the minimum frequency specified in the SSM operational protocol, that compromise SSM spectral acquisition. This optical parameter space has not previously been characterized. We established a standardized bench-top protocol, applied it to a multi-manufacturer panel of 20 flexible ureteroscopes, cystoscopes, and a semirigid ureteroscope, and generated a complete SSM compatibility matrix.

## Materials and methods

### Study design and device inclusion

This prospective bench-top study was conducted at the Endolase lab, PIMM-Arts et Métiers Paris Tech, and Tenon Hospital (Paris, France) between April and May 2026, following a pre-specified bench-top protocol. Endoscope inclusion reflected local availability rather than a pre-defined sampling frame, yielding a convenience cohort from the authors’ unit and collaborating centers. Single-use and reusable flexible ureteroscopes, cystoscopes, and a semirigid ureteroscope were eligible; each scope-videobox pair was characterized as an integrated unit, since the combined system determines delivered illumination. The analysis includes the 20 combinations with complete StoneSense-processed spectral band data.

### Instrumentation

**Fig. 1:**
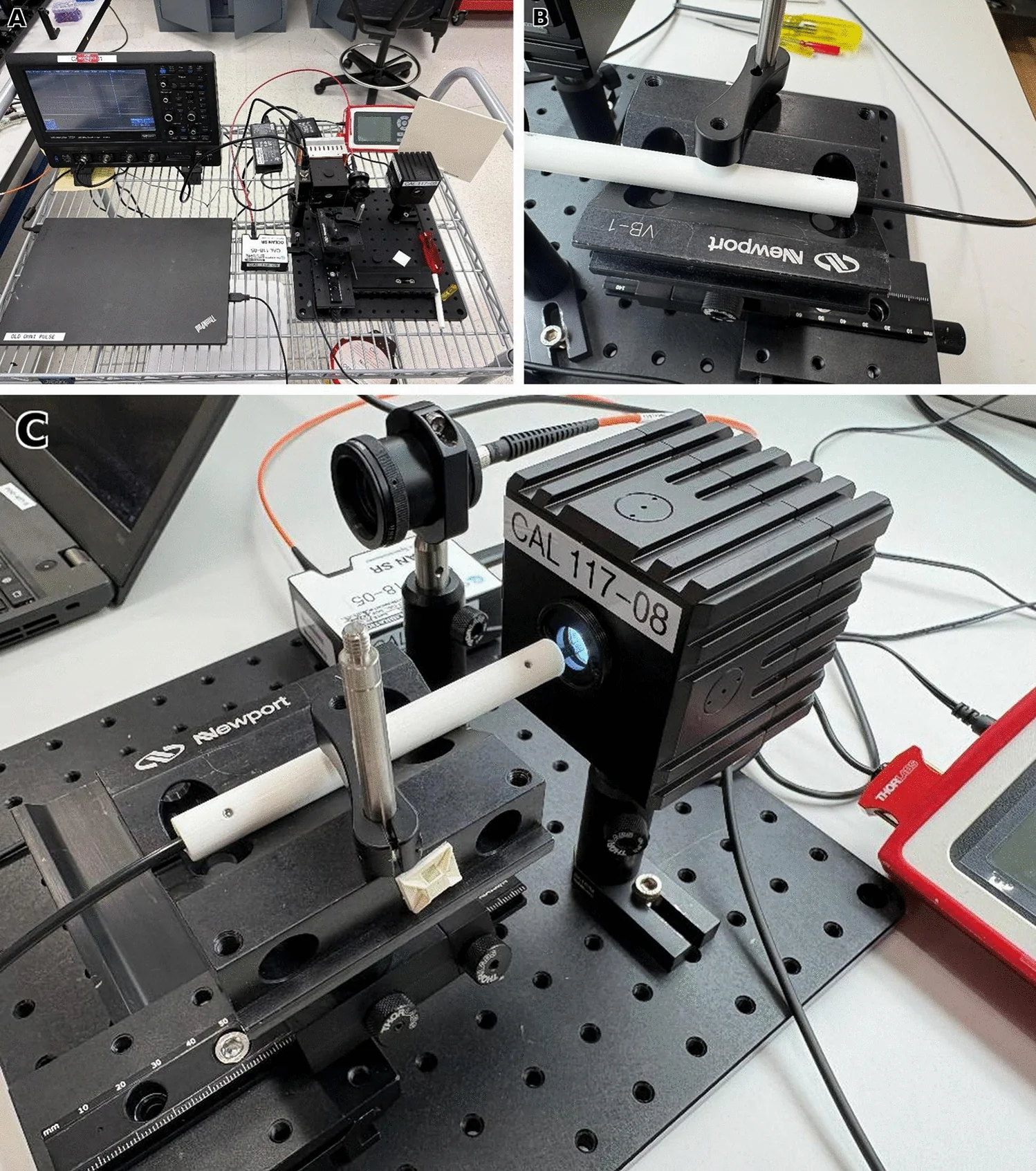
Bench-top optical characterization setup. **A** Complete test bench overview showing the light-tight enclosure, Ocean Optics SR spectrometer, Thorlabs PM100D power meter with integrating sphere, and S142C sensor. **B** Scope distal tip on Newport V-mount for spectral acquisition in free-space configuration. C Scope aimed at the Thorlabs S142C sensor for total optical power measurement

The test bench comprised an Ocean Optics SR Compact Spectrometer (OceanView software, Ocean Optics, Orlando, FL, USA) with correction for absolute power irradiance (Ocean Insight, HL-3 Plus), a Thorlabs PM100D power meter (Newton, NJ, USA) with S142C sensor and integrating sphere for total power measurements, a Thorlabs PDA100A2 photoreceiver and Rigol oscilloscope for temporal characterization, and optical fibers of 150–940 µm core diameter for mechanical compatibility testing (Fig. 1). All devices underwent a minimum one-minute warm-up at Manual brightness before measurement.

### Measurement procedures

Spectral measurements were acquired at minimum, medium, and maximum brightness in a light-tight enclosure with boxcar averaging of 5 and automatic integration time; total power was recorded at 550 nm reference wavelength. Temporal profile was assessed oscilloscopically at 30 dB gain and classified as CW, PWM, or hybrid. Mechanical compatibility was determined by binary pass/fail insertion testing for each fiber size. Band-decomposed power was calculated using the StoneSense proprietary application (IPG Medical Corporation, Marlborough, MA, USA) for three spectral bands (Band 1; Band 2; Band 3; exact center wavelengths and bandwidths are proprietary to the manufacturer and not disclosed), projecting per-fiber band power from free-space data via a geometric coupling factor accounting for fiber numerical aperture, core diameter, and working distance; the underlying coefficients and classification algorithm are proprietary to IPG Medical Corporation and were not disclosed to the investigators. This geometric model does not explicitly account for angular light distribution, lens characteristics, or platform-specific coupling losses, and its accuracy relative to direct in-channel measurement remains a primary target for future validation.

### SSM compatibility criteria

A device-brightness-fiber combination was classified PASS only if all three conditions were met: (1) mechanical compatibility (fiber inserts without obstruction); (2) optical power within range (each band power between the minimum (Supplementary Table 2) and maximum (Supplementary Table 3) thresholds for the corresponding fiber diameter); (3) temporal compatibility (CW or PWM ≥ 2 kHz). Failure mode was recorded as Mechanical, Optical, or Temporal. Criteria (1) and (2) were assessed independently, in separate experimental steps. Throughout this manuscript, "saturation" denotes modeled band power exceeding the predefined SSM upper threshold, not literal saturation of the spectrometer or power meter detectors used for free-space measurement.

### Statistical analysis

Continuous parameters are reported as mean, SD, and range where aggregated, or as single device-level values where each device-videobox combination is a unique unit with no within-device replication. Categorical outcomes are expressed as counts and proportions; no inferential testing was performed, consistent with a descriptive study.

## Results

### Device inventory and temporal profile

The 20 included combinations comprised 15 fURS, 4 cystoscopes, and 1 semirigid ureteroscope from 7 manufacturers (Supplementary table 1); single-use platforms accounted for 55% (11/20). All 20 devices exhibited a CW temporal profile at all brightness levels, conferring unconditional temporal SSM compatibility. This reflects the cohort’s composition: exclusively LED-based single-use and reusable platforms, plus the Olympus legacy CLV-S190/OTV-S190 xenon-free system (device-level detail in Archetypes 1–4, below).

### Mechanical compatibility

Fibers of 150, 200, and 365 µm were compatible with all 20 devices. The 550 µm fiber was mechanically incompatible with the Karl Storz Flex XC and Flex XC1 (2/20, 10% failure). The 940 µm fiber was compatible only with the Karl Storz 11272 C1 and 27002 L; the LithoVue Elite’s narrower working channel (~1.2 mm) excluded only this fiber size, accepting 150–550 µm.

### Spectral band power and total optical power

**Fig. 2:**
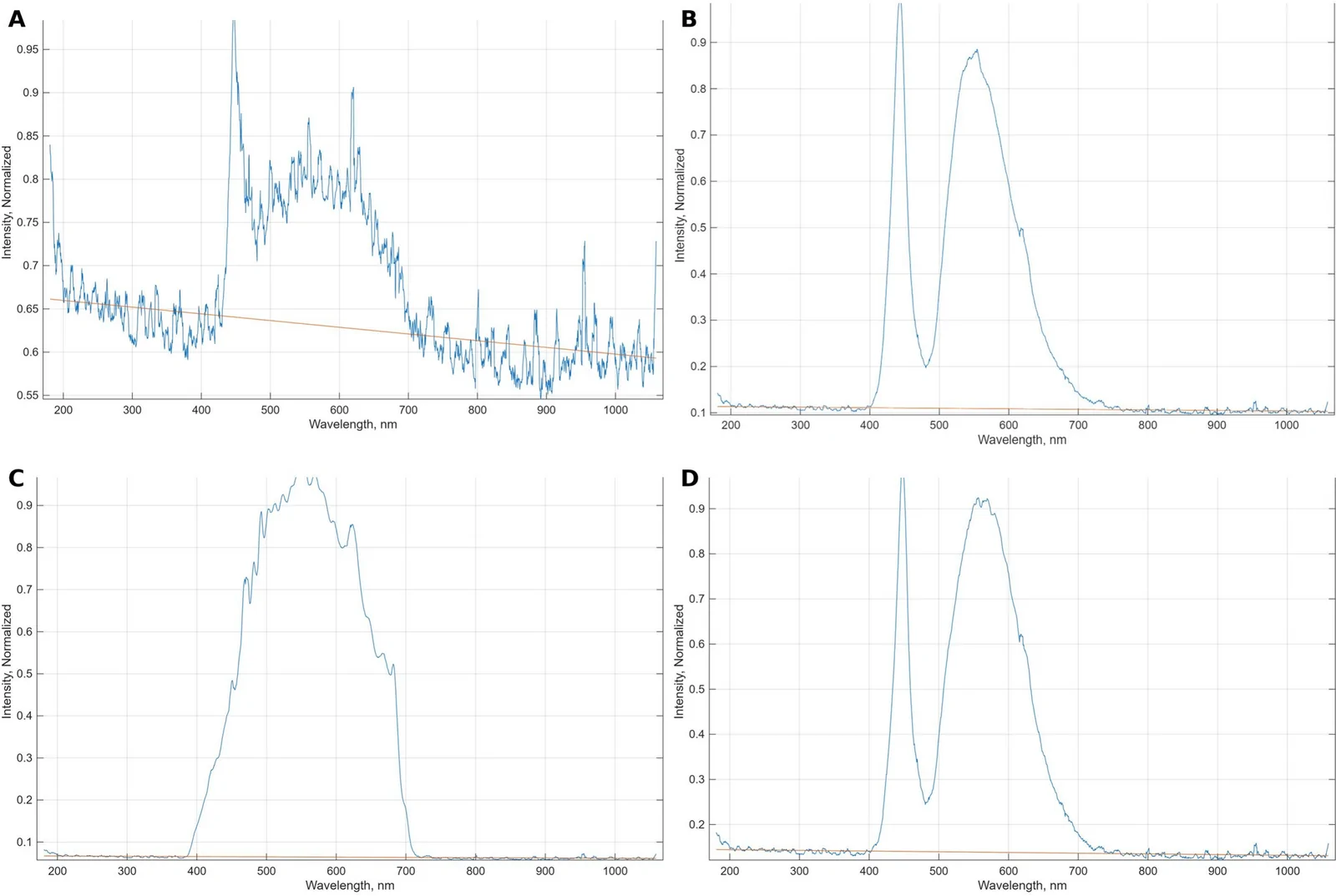
Representative free-space emission spectra (Intensity, Normalized vs. Wavelength, nm) acquired in free-space configuration for each of the four illumination archetypes identified. **A** Archetype 1—Blue-pump LED dominant: Pusen PU3033A 7.5Fr (Band 1 dominant; Band 2 and Band 3 subdominant). **B** Archetype 2—Red-shifted LED/SCB-LED175: Karl Storz Flex X2 (Band 2 ≫ Band 1). **C** Archetype 3—Olympus CLV-S190/OTV-S190 LED system: Olympus URF (Band 1 dominant, intermediate Band 2/Band 1 ratio). **D** Archetype 4—LED-phosphor: Boston Scientific LithoVue Elite (unique spectral inversion: Band 2 and Band 3 exceed Band 1 by factor ≈ 2). Orange line = Spectralon 2% diffuse reflectance reference (dark baseline). Blue line = device emission spectrum

Total free-space optical power at maximum brightness ranged from 3.03 mW (Ambu aScope 5 Uretero, constant across brightness levels) to 60.00 mW (Olympus CYF-VH cystoscope), with the working channel empty. Band-decomposed data are in Supplementary Table 4. Four illumination archetypes were identified (Fig. 2; statistics at maximum brightness). Archetype 1: blue-pump LED dominant (n = 9, single-use and reusable LED platforms): Band 1 exceeded Band 2 in all nine devices; total power 6.15 ± 4.22 mW, Band 2/Band 1 ratio 0.39 ± 0.11. Archetype 2: red-shifted LED/SCB-LED175 (n = 4, Karl Storz H3-link platforms): Band 2 and Band 3 exceeded Band 1 in all four; ratio 2.35 ± 0.54; total power 4.20 mW (Flex X2) to 54.70 mW (Flex XC, constant across settings). Archetype 3: Olympus CLV-S190/OTV-S190 (n = 5, all Olympus URF/CYF platforms): Band 1 dominant at maximum brightness (ratio 0.75 ± 0.07); extremely low output at minimum brightness (mean 100 ± 141 µW) underlies the sub-threshold predictions at this setting. Archetype 4: LED-phosphor (n = 1: LithoVue Elite): Band 2/3 exceeded Band 1 by 2.13-fold, a spectral inversion consistent with LED-phosphor technology [20]; total power constant at 7.26 mW. The Flex XC1 offers a single, fixed brightness setting (reported as Max, the only level the device provides); its ratio (0.44) matched Archetype 1. The Flex XC delivered a constant 54,700 µW regardless of the on-screen brightness setting (see Discussion).

### SSM compatibility assessment

Building on the mechanical findings above, optical threshold criteria, recalculated against the corrected SSM reference thresholds (Supplementary Table 3), were satisfied for the 200 µm and 365 µm fibers in 18 of the 20 devices at medium/maximum brightness; the Karl Storz Flex XC and the Olympus CYF-VH cystoscope exceed the maximum threshold at both fiber sizes and are the two exceptions at these sizes. Further saturation, restricted to the 550 µm and/or 940 µm fiber sizes, was identified for six further devices, most already mechanically restricted or marginal; the complete device-by-fiber matrix is provided in Table 1. The Olympus URF-V, URF-V2, URF-P5, and URF-P6 fell below the minimum threshold at minimum brightness, reflecting the very low output of the CLV-S190/OTV-S190 system at this setting; the URF-V2 additionally fell below the 150 µm minimum threshold at medium brightness.

**Table 1:** Mechanical and optical compatibility by device and optical fiber diameter (medium/maximum brightness; corrected SSM thresholds)

Device	150 µm	200 µm	365 µm	550 µm	940 µm
HugeMed HU 6.3Fr	Mech: ✓ YES **Opt: ✓ PASS**	Mech: ✓ YES **Opt: ✓ PASS**	Mech: ✓ YES **Opt: ✓ PASS**	Mech: ✓ YES **Opt: ✓ PASS**	**Mech: ✗ NO** Opt: –
HugeMed HU30S 7.5Fr	Mech: ✓ YES **Opt: ✓ PASS**	Mech: ✓ YES **Opt: ✓ PASS**	Mech: ✓ YES **Opt: ✓ PASS**	Mech: ✓ YES **Opt: ✓ PASS**	**Mech: ✗ NO** Opt: –
Pusen PU3033A 7.5Fr	Mech: ✓ YES **Opt: ✓ PASS**	Mech: ✓ YES **Opt: ✓ PASS**	Mech: ✓ YES **Opt: ✓ PASS**	Mech: ✓ YES **Opt: ✓ PASS**	**Mech: ✗ NO** Opt: –
Pusen PU3033H asp. 7.5Fr	Mech: ✓ YES **Opt: ✓ PASS**	Mech: ✓ YES **Opt: ✓ PASS**	Mech: ✓ YES **Opt: ✓ PASS**	Mech: ✓ YES **Opt: ✓ PASS**	**Mech: ✗ NO** Opt: –
Macrolux U20-B 7.5Fr	Mech: ✓ YES **Opt: ✓ PASS**	Mech: ✓ YES **Opt: ✓ PASS**	Mech: ✓ YES **Opt: ✓ PASS**	Mech: ✓ YES **Opt: ✓ PASS**	**Mech: ✗ NO** Opt: –
Macrolux U10-B 8.25Fr	Mech: ✓ YES **Opt: ✓ PASS**	Mech: ✓ YES **Opt: ✓ PASS**	Mech: ✓ YES **Opt: ✓ PASS**	Mech: ✓ YES **Opt: ✓ PASS**	**Mech: ✗ NO** Opt: –
Macrolux C50-C Cystoscope	Mech: ✓ YES **Opt: ✓ PASS**	Mech: ✓ YES **Opt: ✓ PASS**	Mech: ✓ YES **Opt: ✓ PASS**	Mech: ✓ YES **Opt: ✗ FAIL**	**Mech: ✗ NO** Opt: –
Ambu aScope 5 Uretero	Mech: ✓ YES **Opt: ✓ PASS**	Mech: ✓ YES **Opt: ✓ PASS**	Mech: ✓ YES **Opt: ✓ PASS**	Mech: ✓ YES **Opt: ✓ PASS**	**Mech: ✗ NO** Opt: –
Ambu aScope 4 Cysto	Mech: ✓ YES **Opt: ✓ PASS**	Mech: ✓ YES **Opt: ✓ PASS**	Mech: ✓ YES **Opt: ✓ PASS**	Mech: ✓ YES **Opt: ✓ PASS**	**Mech: ✗ NO** Opt: –
Karl Storz Flex XC	Mech: ✓ YES **Opt: ✓ PASS**	Mech: ✓ YES **Opt: ✗ FAIL**	Mech: ✓ YES **Opt: ✗ FAIL**	**Mech: ✗ NO** Opt: –	**Mech: ✗ NO** Opt: –
Karl Storz Flex XC1	Mech: ✓ YES **Opt: ✓ PASS**	Mech: ✓ YES **Opt: ✓ PASS**	Mech: ✓ YES **Opt: ✓ PASS**	**Mech: ✗ NO** Opt: –	**Mech: ✗ NO** Opt: –
Karl Storz Flex X2	Mech: ✓ YES **Opt: ✓ PASS**	Mech: ✓ YES **Opt: ✓ PASS**	Mech: ✓ YES **Opt: ✓ PASS**	Mech: ✓ YES **Opt: ✓ PASS**	**Mech: ✗ NO** Opt: –
Karl Storz 11272 C1 (flex. cysto)	Mech: ✓ YES **Opt: ✓ PASS**	Mech: ✓ YES **Opt: ✓ PASS**	Mech: ✓ YES **Opt: ✓ PASS**	Mech: ✓ YES **Opt: ✗ FAIL**	Mech: ✓ YES **Opt: ✗ FAIL**
Karl Storz 27002 L (semirigid)	Mech: ✓ YES **Opt: ✓ PASS**	Mech: ✓ YES **Opt: ✓ PASS**	Mech: ✓ YES **Opt: ✓ PASS**	Mech: ✓ YES **Opt: ✓ PASS**	Mech: ✓ YES **Opt: ✓ PASS**
Olympus URF-V2	Mech: ✓ YES **Opt: ✗ FAIL**	Mech: ✓ YES **Opt: ✓ PASS**	Mech: ✓ YES **Opt: ✓ PASS**	Mech: ✓ YES **Opt: ✓ PASS**	**Mech: ✗ NO** Opt: –
Olympus URF-V	Mech: ✓ YES **Opt: ✓ PASS**	Mech: ✓ YES **Opt: ✓ PASS**	Mech: ✓ YES **Opt: ✓ PASS**	Mech: ✓ YES **Opt: ✗ FAIL**	**Mech: ✗ NO** Opt: –
Olympus URF-P5	Mech: ✓ YES **Opt: ✓ PASS**	Mech: ✓ YES **Opt: ✓ PASS**	Mech: ✓ YES **Opt: ✓ PASS**	Mech: ✓ YES **Opt: ✓ PASS**	**Mech: ✗ NO** Opt: –
Olympus URF-P6	Mech: ✓ YES **Opt: ✓ PASS**	Mech: ✓ YES **Opt: ✓ PASS**	Mech: ✓ YES **Opt: ✓ PASS**	Mech: ✓ YES **Opt: ✗ FAIL**	**Mech: ✗ NO** Opt: –
Olympus CYF-VH Cystoscope	Mech: ✓ YES **Opt: ✓ PASS**	Mech: ✓ YES **Opt: ✗ FAIL**	Mech: ✓ YES **Opt: ✗ FAIL**	Mech: ✓ YES **Opt: ✗ FAIL**	**Mech: ✗ NO** Opt: –
BS LithoVue Elite	Mech: ✓ YES **Opt: ✓ PASS**	Mech: ✓ YES **Opt: ✓ PASS**	Mech: ✓ YES **Opt: ✓ PASS**	Mech: ✓ YES **Opt: ✗ FAIL**	**Mech: ✗ NO** Opt: –

Olympus URF-V2, URF-V, URF-P5, and URF-P6 additionally fall below the minimum optical threshold at minimum brightness for all mechanically compatible fiber sizes (150–550 µm); URF-V2 falls below the 150 µm minimum threshold at medium brightness as well. These minimum-brightness limitations are not reflected in the PASS/FAIL grading above, which follows the medium/maximum operating recommendation

*Mech* mechanical fiber compatibility (insertion without obstruction), *Opt* optical band-power compatibility at medium and/or maximum brightness against the corrected SSM thresholds (Supplementary Table 2–Supplementary Table 3), *PASS* compatible, *FAIL* incompatible (mechanical obstruction, or optical sub-threshold/saturation—see Supplementary Table 4 for underlying values)

Minimum-brightness-only sub-threshold findings (Olympus URF-family, 4 devices) are noted separately below the table and are not scored as FAIL here, consistent with the medium/maximum operating recommendation. For standard ureteroscopic lithotripsy, the 200 µm and 365 µm columns are the most clinically relevant: all platforms PASS at both sizes except the Karl Storz Flex XC and Olympus CYF-VH

## Discussion

This study presents the first systematic bench-top characterization of flexible ureteroscopes and cystoscopes against predefined SSM optical compatibility criteria, producing a device-level pass/fail matrix across five fiber sizes and three brightness levels. This work sits within a broader effort to automate stone-tissue discrimination, pioneered through autofluorescence-based systems [14, 15] and more recently extended to clinical DRS platforms [16, 17]. The central finding, detailed above, is that the 200 µm and 365 µm fibers — the sizes most widely used in clinical laser lithotripsy [3, 5] — are predicted compatible in 18 of the 20 devices, with the Flex XC and CYF-VH as exceptions. Failures are otherwise restricted to predictable, architecture-driven scenarios: sub-threshold power at minimum brightness in legacy low-output platforms, and band saturation in high-power devices with the larger (550/940 µm) fibers. All devices are temporally compatible (CW), simplifying deployment relative to Olympus xenon-lamp platforms with hybrid PWM/CW profiles to be reported separately.

As emphasized above, per-fiber band power values are StoneSense-modeled projections, not direct fiber-in-channel measurements; mechanical compatibility was assessed separately. The modeled incompatibilities are mechanistically predictable from device illumination architecture: sub-threshold values at minimum brightness for the Olympus CLV-S190/OTV-S190 series reflect their very low luminance at the lowest setting (14–380 µW) — clinically minor, as minimum brightness is rarely used during lithotripsy [18]. Saturation in the Flex XC for the 200 µm and 365 µm fibers at all brightness levels results from its constant 54,700 µW output, whose brightness controls modulate display gain rather than illumination power; the SSM is therefore predicted incompatible with the Flex XC at these fiber sizes at any setting. Saturation for the Olympus CYF-VH with the 200 µm and 365 µm fibers at maximum brightness is predicted avoidable at medium brightness; saturation at 550 µm, however, persists even at medium brightness given this device’s very high total optical output.

The four illumination archetypes identified have implications beyond threshold compliance. The LED-phosphor profile of the LithoVue Elite, Bands 2/3 exceeding Band 1 by ~2-fold, inverse of conventional blue-pump LED sources, represents a qualitatively distinct spectral input to the SSM spectrometer. Although modeled band power values fall within thresholds, the impact of this inverted profile on SSM classification accuracy requires dedicated prospective validation in situ [20]. The red-shifted LED archetype of Karl Storz H3-link platforms (Flex X2, 11272 C1, 27002 L) also meets modeled thresholds but may interact differently with the classification algorithm relative to blue-dominant sources [14–16]; this possibility is speculative, as no stone/tissue classification experiments were performed in this study. According to the manufacturer, the SSM classification algorithm has been designed to be independent of these spectral differences; independent validation of this claim is outside the scope of the present optical characterization.

Cystoscope inclusion extends SSM characterization to bladder stone management [1, 24]. All three tested flexible cystoscopes show modeled band power within thresholds for 200 µm and 365 µm fibers at medium brightness. The Karl Storz 11272 C1 and semirigid 27002 L are the only platforms with 940 µm fiber mechanical compatibility; with the corrected thresholds, only the 27002 L retains modeled optical compatibility at this fiber diameter, as the 11272 C1 now exceeds the maximum threshold, relevant to aspiration-lithotripsy workflows.

Practically, operators intending to use StoneSense should select a 200 µm or 365 µm fiber where appropriate and avoid minimum brightness on Olympus CLV-S190/OTV-S190 platforms, where output falls below the modeled detection threshold; medium and maximum brightness are otherwise interchangeable. A key caveat, illustrated by the Flex XC, is that on-screen “brightness” does not always reflect actual illumination: some processors adjust digital image gain rather than the light source, so a visually dimmer image does not guarantee lower emitted power. Operators should not infer compatibility from displayed brightness alone. If automatic brightness regulation is active during a procedure, the illumination feeding StoneSense may change unnoticed; whether this affects real-time compatibility was not tested and warrants specific evaluation. These fiber-diameter recommendations concern SSM optical compatibility only, not laser energy or pulse-frequency settings, which follow established, StoneSense-independent lithotripsy protocols not addressed here.

Several limitations apply. First, the analysis is restricted to 20 combinations with available StoneSense data; 21 excluded Olympus xenon-lamp combinations were precisely those where a hybrid PWM/CW profile would make the temporal criterion clinically relevant, so the finding of unconditional temporal compatibility reflects cohort composition rather than a generalizable result. Second, bench-top output may differ in vivo due to scope deflection, lens soiling, and light source aging [18]; similarly, mechanical testing used static fiber insertion on an unpowered, undeflected scope and does not capture deflection, friction, or distal positioning effects during active use. Finally, optical degradation of reusable scopes over their lifetime may shift compatible combinations toward minimum thresholds [23]. Formal uncertainty propagation was not performed, as each combination was measured once by design; instrument-level uncertainty is bounded by manufacturer calibration tolerances, but uncertainty from the undisclosed proprietary coupling model cannot be quantified. Findings near threshold boundaries should be interpreted with this caution.

## Conclusions

This bench-top protocol characterized 20 device-videobox combinations from seven manufacturers against predefined SSM optical criteria, yielding modeled band-power projections rather than direct fiber-in-channel measurements. The 200 µm and 365 µm fibers at medium/maximum brightness are predicted to meet SSM thresholds across 18 of the 20 platforms, with the Karl Storz Flex XC and the Olympus CYF-VH cystoscope as the two exceptions owing to their high-power output; these model-based predictions should guide prioritization of in-channel validation studies, not immediate changes to clinical practice, and the remaining incompatibilities are otherwise device-architecture-driven and addressable through fiber and brightness selection. All 20 devices exhibited CW emission, conferring unconditional temporal SSM compatibility within this cohort. These results provide a first-step compatibility reference for SSM deployment planning; direct experimental validation with the fiber in the working channel is required before clinical implementation. StoneSense remains pre-clinical: this compatibility work and the ongoing in vitro discrimination study noted above are intended to precede first-in-human evaluation, not yet begun.

## Supplementary Information

Below is the link to the electronic supplementary material.

Supplementary file1 (DOCX 45 KB)

## Data Availability

Raw spectral measurements (total free-space optical power, normalized emission spectra) and per-band power data are available from the corresponding author upon reasonable request. StoneSense-modeled per-fiber band power projections are derived from a proprietary algorithm developed by IPG Medical Corporation and cannot be independently shared by the authors.

## References

[1] Scales CD Jr, Smith AC, Hanley JM, Saigal CS, Urologic Diseases in America Project (2012) Prevalence of kidney stones in the United States. Eur Urol 62(1):160–5. 10.1016/j.eururo.2012.03.052 PMC3362665 22498635

[2] Sorokin I, Mamoulakis C, Miyazawa K, Rodgers A, Talati J, Lotan Y (2017) Epidemiology of stone disease across the world. World J Urol 35(9):1301–1320. 10.1007/s00345-017-2008-6 28213860

[3] Türk C, Neisius A, Petřík A, Seitz C, Skolarikos A, Thomas K, et al. EAU Guidelines on Urolithiasis. Arnhem: European Association of Urology; 2023. Available from: https://uroweb.org/guidelines/urolithiasis

[4] Dauw CA, Simeon L, Alruwaily AF, Sana MF, Hollingsworth JM, Roberts WW et al (2015) Contemporary practice patterns of flexible ureteroscopy for treating renal stones: results of a worldwide survey. J Endourol 29(11):1221–30. 10.1089/end.2015.0260 26154856

[5] Kronenberg P, Traxer O (2014) In vitro fragmentation efficiency of holmium:yttrium-aluminum-garnet (YAG) laser lithotripsy. BJU Int 114(2):261–7. 10.1111/bju.12538 24219145

[6] Enikeev D, Taratkin M, Klimov R, Alyaev Y, Rapoport L, Gazimiev M et al (2020) Thulium-fiber laser for lithotripsy: first clinical experience in percutaneous nephrolithotomy. World J Urol 38(12):3069–3074. 10.1007/s00345-020-03134-x 32108256

[7] Panthier F, Doizi S, Corrales M, Traxer O (2022) Comparison of in-vitro stone ablation rates and dusting ability of the SuperPulsed thulium fiber laser and a high-power holmium:YAG laser. World J Urol 40(1):225–231. 10.1007/s00345-021-03821-9

[8] Natalin RA, Varble N, Lukasiewicz A, Elsamra SE, Duty B, Kavoussi L et al (2022) Evaluation of ureteroscopy-related complications using a real-world cohort. J Endourol 36(8):1055–1061. 10.1089/end.2021.0808

[9] Scotland KB, Shim I, Hajiha M, Honey RJD (2023) Ureteral injury from flexible ureteroscopy: predictors. J Endourol 37(2):207–213. 10.1089/end.2022.0455

[10] Lange B, Jocham D, Brinkmann R, Cordes J (2014) Stone/tissue differentiation during intracorporeal lithotripsy using diffuse white light reflectance spectroscopy: in vitro and clinical measurements. Lasers Surg Med 46(8):614–619. 10.1002/lsm.22276 25130717

[11] Schütz J, Miernik A, Brandenburg A, Schlager D (2019) Experimental evaluation of human kidney stone spectra for intraoperative stone-tissue-instrument analysis using autofluorescence. J Urol 201(1):182–187. 10.1016/j.juro.2018.07.067 10.1016/j.juro.2018.07.067 30077558

[12] Lange B, Jocham D, Brinkmann R, Cordes J (2017) Stone/tissue differentiation for holmium laser lithotripsy using autofluorescence: clinical proof of concept study. Lasers Surg Med 49(4):361–365. 10.1002/lsm.22611 27859390

[13] Lange B, Cordes J, Brinkmann R (2020) Exploiting the aiming beam to increase the safety of laser lithotripsy: experimental evaluation of light reflection and fluorescence. Lasers Surg Med 52(5):456–471. 10.1002/lsm.23170 31512270

[14] Schlager D, Miernik A, Lamrini S, Schütz J, Brandenburg A, Hein S et al (2019) A novel laser lithotripsy system with automatic real-time urinary stone recognition: computer controlled ex vivo lithotripsy is feasible and reproducible. J Urol 202(6):1263–1269. 10.1097/JU.0000000000000360 31347954

[15] Schlager D, Schulte A, Schütz J, Brandenburg A, Schell C, Lamrini S et al (2021) Laser-guided real-time automatic target identification for endoscopic stone lithotripsy: a two-arm in vivo porcine comparison study. World J Urol 39(7):2719–2726. 10.1007/s00345-020-03446-y PMC8332575 32960325

[16] Korneva N, Budylin G, Tseregorodtseva P, Nikitin D, Andreeva V, Baytsaeva O et al (2026) Optical feedback for safe automatic laser lithotripsy: tissue sensor implementation in a clinical system. Lasers Surg Med 58:38–48. 10.1002/lsm.70081 41398708

[17] Lange B, Ozimek T, Wießmeyer JR, Kramer MW, Merseburger AS, Brinkmann R (2025) Fluorescence-guided laser lithotripsy: first experience from a clinical study with automatic blocking of the treatment laser beam. Transl Biophotonics 7:e70002. 10.1002/tbio.70002

[18] Soria F, Haifler M, Srivastava A, Doizi S, Traxer O (2020) Evolution of flexible ureterorenoscopes: from optical to digital and from reusable to single-use. Transl Androl Urol 9(Suppl 2):S125–S134. 10.21037/tau.2019.11.16

[19] Lima A, Reeves T, Geraghty R, Somani BK (2020) Impact of digital ureteroscopy: a systematic review and update. Curr Urol Rep 21(11):47. 10.1007/s11934-020-01001-5

[20] Kim JK, Watanabe H, Yamada Y, Im SH, Hollenbeck S, Chung WH (2020) Phosphor-converted LED illumination for color quality and photometric performance. Opt Express 28(3):3734–3748. 10.1364/OE.378819

[21] Doizi S, Traxer O (2018) Flexible ureteroscopy: technique, tips and tricks. Urolithiasis 46(1):47–58. 10.1007/s00240-017-1020-3 29222575

[22] Assimos D, Krambeck A, Miller NL, Monga M, Murad MH, Nelson CP et al (2016) Surgical management of stones: AUA/Endourological Society joint guideline, part II. J Urol 196(4):1161–9. 10.1016/j.juro.2016.05.091 27238615

[23] Mager R, Kurosch M, Höfner T, Fath-Mobarakeh K, Haferkamp A, Herrmann T (2018) Clinical outcomes and costs of reusable and single-use digital flexible ureterorenoscopes: a prospective cohort study. Urolithiasis 46(6):587–593. 10.1007/s00240-017-1017-y 29356873

[24] Traxer O, Zorzi F, Yaroslavsky IV, Kovalenko A, Andreeva V, Childs J et al (2026) Pre-clinical evaluation of a laser system with emission control based on stone vs soft tissue recognition. BJU Int. 10.1111/bju.70301 PMC13371826 42219295

[25] Morozov A, Matkovskiy I, Somani B, Baniel J, Traxer O, Lifshitz D et al (2025) Pre-clinical evaluation of laser system with emission control based on stone vs soft tissue recognition. J Urol 213(5 Suppl):e627. 10.1097/01.JU.0001110072.52045.9d.15

