# Supplementary material for: Optical characterization of endoscopes for StoneSense™ compatibility
Source: World J Urol. 2026 Nov 2;44(1):778. doi: 10.1007/s00345-026-06845-9 (PMC13727889; doi:10.1007/s00345-026-06845-9)
Supplement: Supplementary file 1 [file 345_2026_6845_MOESM1_ESM.docx]

**Supplementary Table 1.** *Device inventory — 20 combinations with complete spectral band data.*

| **#** | **Scope model** | **Manufacturer** | **Video box / illumination** | **Type** | **Code** |
| --- | --- | --- | --- | --- | --- |
| **1** | HU 6.3 Fr | HugeMed | HugeMed | fURS (single-use) | **HUG** |
| **2** | HU30S 7.5 Fr | HugeMed | HugeMed | fURS (single-use) | **30S** |
| **3** | PU3033A 7.5 Fr | Pusen | PUSEN PV300 | fURS (single-use) | **PU3** |
| **4** | PU3033H 7.5 Fr aspiration | Pusen | PUSEN | fURS (single-use) | **DIS** |
| **5** | U20-B 7.5 Fr | Macrolux/Sophysa | Viewhub Macrolux S13-T | fURS | **U20** |
| **6** | U10-B 8.25 Fr | Macrolux/Sophysa | Viewhub Macrolux S13-T | fURS | **U10** |
| **7** | C50-C (14.4 Fr cystoscope) | Macrolux/Sophysa | Viewhub Macrolux S13-T | Cystoscope | **C50** |
| **8** | aScope 5 Uretero 8.1 Fr | Ambu | Ambu aBox 2 | fURS (single-use) | **ASC** |
| **9** | aScope 4 Cysto | Ambu | Ambu aView 2 Advance | Cystoscope (single-use) | **ACS** |
| **10** | Flex XC | Karl Storz | Image 1S X-link | fURS | **SXC** |
| **11** | Flex XC1 | Karl Storz | Image 1S X-link | fURS | **XC1** |
| **12** | Flex X2 | Karl Storz | Image 1S H3-link + SCB-LED175 | fURS | **X2** |
| **13** | 11272 C1 | Karl Storz | Image 1S H3-link + SCB-LED175 | Cystoscope | **CC1** |
| **14** | 27002 L (semirigid URS) | Karl Storz | Image 1S H3-link + SCB-LED175 | Semirigid URS | **270** |
| **15** | URF-V2 | Olympus | CLV-S190 / OTV-S190 | fURS | **URF-V2** |
| **16** | URF-V | Olympus | CLV-S190 / OTV-S190 | fURS | **URV** |
| **17** | URF-P5 | Olympus | CLV-S190 / OTV-S190 | fURS | **URP** |
| **18** | URF-P6 | Olympus | CLV-S190 / OTV-S190 | fURS | **URF-P6** |
| **19** | CYF-VH (flexible cystoscope) | Olympus | CLV-S190 / OTV-S190 | Cystoscope | **CYF** |
| **20** | LithoVue Elite | Boston Scientific | Stone smart connect | fURS (single-use) | **LTE** |

**Supplementary Table 2.** *Minimum spectral band power thresholds (µW) per fiber diameter.*

*Spectralon 2%; counts = 50.*

| **Fiber Diameter (µm)** | **Band 1** | **Band 2** | **Band 3** | **Sum (µW)** |
| --- | --- | --- | --- | --- |
| **150 µm** | 24 | 13 | 10 | **47** |
| **200 µm** | 8 | 4 | 3 | **15** |
| **365 µm** | 4 | 2 | 2 | **8** |
| **550 µm** | 1 | 1 | 1 | **3** |
| **940 µm** | 2 | 1 | 1 | **4** |

**Supplementary Table 3.** *Maximum spectral band power thresholds (µW) per fiber diameter.*

*Spectralon 99%; counts = 3,500,000 (corrected calibration reference).*

| **Fiber Diameter (µm)** | **Band 1** | **Band 2** | **Band 3** |
| --- | --- | --- | --- |
| **150 µm** | 27,682 | 14,789 | 10,500 |
| **200 µm** | 10,367 | 5,300 | 3,789 |
| **365 µm** | 5,265 | 2,761 | 1,924 |
| **550 µm** | 1,510 | 894 | 633 |
| **940 µm** | 2,386 | 1,677 | 1,221 |

**Supplementary Table 4.** *Spectral band power (µW) at three brightness levels for all included devices.*

*All devices: CW temporal profile. “All” = output confirmed constant across all three nominal brightness settings (dial has no effect on emitted power). ‡ = device provides only a single, fixed brightness setting (no Min/Med control exists). See Table 1 for the complete per-fiber optical/mechanical compatibility matrix.*

| **Code** | **Level** | **Total P (µW)** | **Band 1** | **Band 2** | **Band 3** | **Device** |
| --- | --- | --- | --- | --- | --- | --- |
| **HUG** | Min | 560 | 52.1 | 10.5 | 9 | HugeMed HU 6.3Fr |
|  | Med | 3,460 | 406 | 154 | 130 |  |
|  | Max | 5,080 | 626 | 256 | 218 |  |
| **30S** | Min | 540 | 46 | 9.1 | 8.2 | HugeMed HU30S 7.5Fr |
|  | Med | 3,480 | 333 | 135 | 123 |  |
|  | Max | 5,170 | 512 | 233 | 212 |  |
| **PU3** | Min | 899 | 75 | 19.2 | 17 | Pusen PU3033A 7.5Fr |
|  | Med | 2,350 | 202 | 82.4 | 73.3 |  |
|  | Max | 3,550 | 309 | 113 | 99.6 |  |
| **DIS** | Min | 870 | 75.4 | 17.5 | 14.9 | Pusen PU3033H asp. 7.5Fr |
|  | Med | 2,240 | 208 | 74.1 | 61.5 |  |
|  | Max | 3,420 | 331 | 139 | 115 |  |
| **U20** | Min | 1,490 | 163 | 44.2 | 35.9 | Macrolux U20-B 7.5Fr |
|  | Med | 2,380 | 281 | 89.3 | 72.4 |  |
|  | Max | 4,100 | 523 | 187 | 151 |  |
| **U10** | Min | 2,600 | 242 | 102 | 85.3 | Macrolux U10-B 8.25Fr |
|  | Med | 4,380 | 420 | 210 | 195 |  |
|  | Max | 7,200 | 710 | 400 | 333 |  |
| **C50** | Min | 5,650 | 798 | 305 | 256 | Macrolux C50-C Cystoscope |
|  | Med | 9,910 | 1,501 | 624 | 523 |  |
|  | Max | 16,630 | 2,632 | 1,161 | 973 |  |
| **ASC** | All† | 3,030 | 761 | 149 | 138 | Ambu aScope 5 Uretero |
| **ACS** | All† | 7,160 | 1,169 | 529 | 495 | Ambu aScope 4 Cysto |
| **SXC** | All | 54,700 | 4,370 | 6,288 | 6,032 | Karl Storz Flex XC |
| **XC1** | Max‡ | 17,000 | 2,549 | 1,128 | 955 | Karl Storz Flex XC1 |
| **X2** | Min | 500 | 31.1 | 34.2 | 28.7 | Karl Storz Flex X2 |
|  | Med | 2,790 | 111 | 309 | 267 |  |
|  | Max | 4,200 | 178 | 476 | 410 |  |
| **CC1** | Min | 2,550 | 143 | 208 | 172 | Karl Storz 11272 C1 (flex. cysto) |
|  | Med | 14,370 | 547 | 1,522 | 1,253 |  |
|  | Max | 21,800 | 920 | 2,276 | 1,879 |  |
| **270** | Min | 770 | 48.4 | 48.7 | 42.3 | Karl Storz 27002 L (semirigid) |
|  | Med | 4,300 | 188 | 465 | 406 |  |
|  | Max | 6,400 | 280 | 793 | 605 |  |
| **URF-V2** | Min | 16 | 1.7 | 0.9 | 0.8 | Olympus URF-V2 |
|  | Med | 500 | 42.1 | 6.2 | 5.4 |  |
|  | Max | 3,490 | 438 | 296 | 269 |  |
| **URV** | Min | 60 | 4.9 | 0.7 | 0.6 | Olympus URF-V |
|  | Med | 1,350 | 137 | 72.3 | 67.3 |  |
|  | Max | 9,650 | 1,150 | 806 | 741 |  |
| **URP** | Min | 14 | 1.1 | 0.1 | 0.12 | Olympus URF-P5 |
|  | Med | 700 | 75 | 40.3 | 36.3 |  |
|  | Max | 3,650 | 445 | 310 | 280 |  |
| **URF-P6** | Min | 30 | 2.5 | 0.4 | 0.37 | Olympus URF-P6 |
|  | Med | 2,050 | 222 | 170 | 160 |  |
|  | Max | 10,000 | 1,128 | 977 | 925 |  |
| **CYF** | Min | 380 | 32.9 | 7.8 | 7 | Olympus CYF-VH Cystoscope |
|  | Med | 9,300 | 1,022 | 785 | 744 |  |
|  | Max | 60,000 | 7,067 | 5,603 | 5,268 |  |
| **LTE** | Min | 5,170 | 286 | 537 | 530 | BS LithoVue Elite |
|  | Med | 6,270 | 340 | 652 | 646 |  |
|  | Max | 7,260 | 370 | 786 | 776 |  |
